# One-Pot Isothermal LAMP-CRISPR-Based Assay for Klebsiella pneumoniae Detection

**DOI:** 10.1128/spectrum.01545-22

**Published:** 2022-07-20

**Authors:** Xiaotong Qiu, Xueping Liu, Xiao Ma, Ruixue Wang, Shenglin Chen, Fang Li, Zhenjun Li

**Affiliations:** a State Key Laboratory for Infectious Disease Prevention and Control, National Institute for Communicable Disease Control and Prevention, Chinese Center for Disease Control and Prevention, Beijing, China; b School of Laboratory Medicine and Life Sciences, Wenzhou Medical University, Wenzhou, China; c Department of Clinical Laboratory, Beijing Friendship Hospital, Capital Medical University, Beijing, China; d Shanxi Bethune Hospital, Shanxi Academy of Medical Sciences, Tongji Shanxi Hospital, Third Hospital of Shanxi Medical University, Taiyuan, China; e School of Public Health, Shanxi Medical University, Taiyuan, China; f Department of Medicine, Tibet University, Lhasa, China; Johns Hopkins Hospital

**Keywords:** *Klebsiella pneumoniae*, LAMP, CRISPR, Cas12b, CRISPR-top, accurate diagnosis, nucleic acid detection

## Abstract

Klebsiella pneumoniae (K. pneumoniae) is one of the most common pathogens causing nosocomial infection. A rapid, accurate, and convenient detection method is required for early diagnosis and directed therapy of K. pneumoniae infection. CRISPR-top (CRISPR-mediated testing in one pot) is a LAMP-CRISPR-based nucleic acid detection platform, which integrates target preamplification with CRISPR/Cas12b-based detection into a one-pot reaction mixture, performed at a constant temperature. In this study, we established the K. pneumoniae CRISPR-top assay to precisely identify K. pneumoniae at 56°C within 60 min. The reaction mixture with 0.53 μM (each) FIP and BIP, 0.27 μM LF, 0.13 μM (each) F3 and B3, and 2 μM ssDNA fluorescence probe was determined as the optimal reaction system of our assay. The limit of detection of this assay is 1 pg genomic DNA (equivalent to 160 K. pneumoniae cells and 1.6 × 10^5^ CFU/mL for samples) per reaction, which is 10-fold more sensitive than LAMP. Up to 105 strains composed of K. pneumoniae clinical isolates and non-K. pneumoniae strains were correctly identified by our assay. A total of 58 sputum samples collected from patients with respiratory symptoms were used to evaluate the diagnostic performance of the K. pneumoniae CRISPR-top assay. As a result, the K. pneumoniae CRISPR-top assay yielded 100% (33/33) specificity and 96% (24/25) sensitivity, as well as a positive predictive value of 100% (24/24) and a negative predictive value of 97.1% (33/34), which were all higher than LAMP detection. In conclusion, the K. pneumoniae CRISPR-top assay developed in this study is a simple, rapid and ultra-specific method to detect K. pneumoniae.

**IMPORTANCE** Klebsiella pneumoniae is a significant threat to global health. At present, the methods of K. pneumoniae detection are culture-based and instrument-dependent and are not suitable for rapid diagnostic. This study reports K. pneumoniae CRISPR-top assay, which can precisely identify K. pneumoniae using nucleic acids of pure cultures or clinical samples in one pot with one fluid-handling step. The K. pneumoniae CRISPR-top reaction can be completed within 60 min at a constant temperature, thus specific instruments are not required. Our results show that CRISPR-top assay yields enormous advantages compared with LAMP detection. The K. pneumoniae CRISPR-top assay can be a high-efficiency alternative tool for rapid and accurate diagnosis of K. pneumoniae infection, especially in resource-limited settings.

## INTRODUCTION

Klebsiella pneumoniae (K. pneumoniae), a Gram-negative bacillus, is one of the most common bacterial pathogens causing acute respiratory infections and even pneumonia, especially nosocomial acquired infection (1, 2). A nationwide prospective surveillance in China between 2009–2019 revealed K. pneumoniae was one of the three leading bacterial pathogens (i.e., Streptococcus pneumoniae, Mycoplasma pneumoniae, and K. pneumoniae) in patients with acute respiratory infections and was the third (older people) to the fifth (children) causes of bacterial pneumonia (3). In recent years, with multidrug-resistant isolates and hypervirulent variants emerging, *K. pneumonia* has become a significant threat to global public health (4, 5). In some countries and regions, the K. pneumoniae population is highly diverse and the resistant strains have spread across the country over a few years (6). Therefore, a rapid, accurate and convenient detection method is required for early diagnosis and directed therapy of K. pneumoniae infection.

At present, some phenotypic-based identification methods, such as biochemical identification and bacterial identification system, are used to detect K. pneumoniae (7, 8). Matrix-assisted laser desorption/ionization time of flight mass spectrometry (MALDI-TOF MS) was also commonly used to identify K. pneumoniae (9, 10). However, these methods are culture-based, which resulted in a longer diagnosis time. Nucleic acid detection is the key to diagnose early and rapidly control the spread of infectious diseases. Some PCR-based molecular diagnostic techniques have been developed to detect K. pneumoniae and shown a satisfactory effect (11, 12), but expensive precision instruments are required to execute these tests. Moreover, the relatively long reaction time (2–4 h) is another limitation of PCR-based methods. Isothermal amplification is an ideal technology to overcome the disadvantages of PCR-based methods. The loop-mediated isothermal amplification (LAMP) has been widely used in pathogen detection (13, 14). However, a relatively high rate of false-positive results is a shortcoming of the LAMP method (15).

In recent years, several cluster regularly interspaced short palindromic repeats/CRISPR-associated (CRISPR/Cas) based detection platform have been developed and showed high sensitivity and ultra-specificity (16). For instance, CRISPR/Cas12a-based DNA endonuclease targeted CRISPR trans reporter (DETECTR) and one-hour low-cost multipurpose highly efficient system (HOLMES), as well as CRISPR/Cas13a-based specific high-sensitivity enzymatic reporter unlocking (SHERLOCK) assay generated single-base mismatch specificity and attomolar sensitivity in multiple pathogens detection (16–18). However, up to now, most CRISPR-based detection assays consisted of two separate steps, including a) preamplification of the target sequences using amplification techniques (e.g., PCR, loop-mediated isothermal amplification, and recombinase polymerase amplification), and b) detection of the preamplicons using the trans-cleavage activity of the Cas protein. These two-step CRISPR assays require multiple manual operations and thus complicate the detection procedures. Additionally, due to the uncapped operation of preamplification tubes, the risk of cross-contamination in the detection step is increased. Despite some microfluidic equipment and reversible valve-assisted chips developed to decrease the risk of cross-contamination (19, 20), the use of these devices increases economic cost and thus their application is limited. The Cas12aVDet platform and the opvCRISPR platform improved CRISPR-assisted in-tube detection without specific equipment, but a centrifugation and mixing step was still required (21, 22). More recently, Li et al. (23) devised a novel CRISPR-based detection technique named CRISPR-top (CRISPR-mediated testing in one pot), which combined loop-mediated isothermal amplification (LAMP) with CRISPR/Cas12b-based detection in one tube and only required one fluid-handling step. The CRISPR-top assay was applied to detect severe acute respiratory syndrome coronavirus 2 (SARS-CoV-2) and showed high specificity and sensitivity. The whole process of the COVID-19 CRISPR-top assay can be completed within 60 min and the results can be read out by visual fluorescence or lateral flow biosensors. It is worth note that a protospacer adjacent motif (PAM) site is introduced to the target sequence by the engineered forward inner primer (FIP), thus the CRISPR-top assay can be used to detect any sequence even if there is no suitable PAM site in the target.

**FIG 1:**
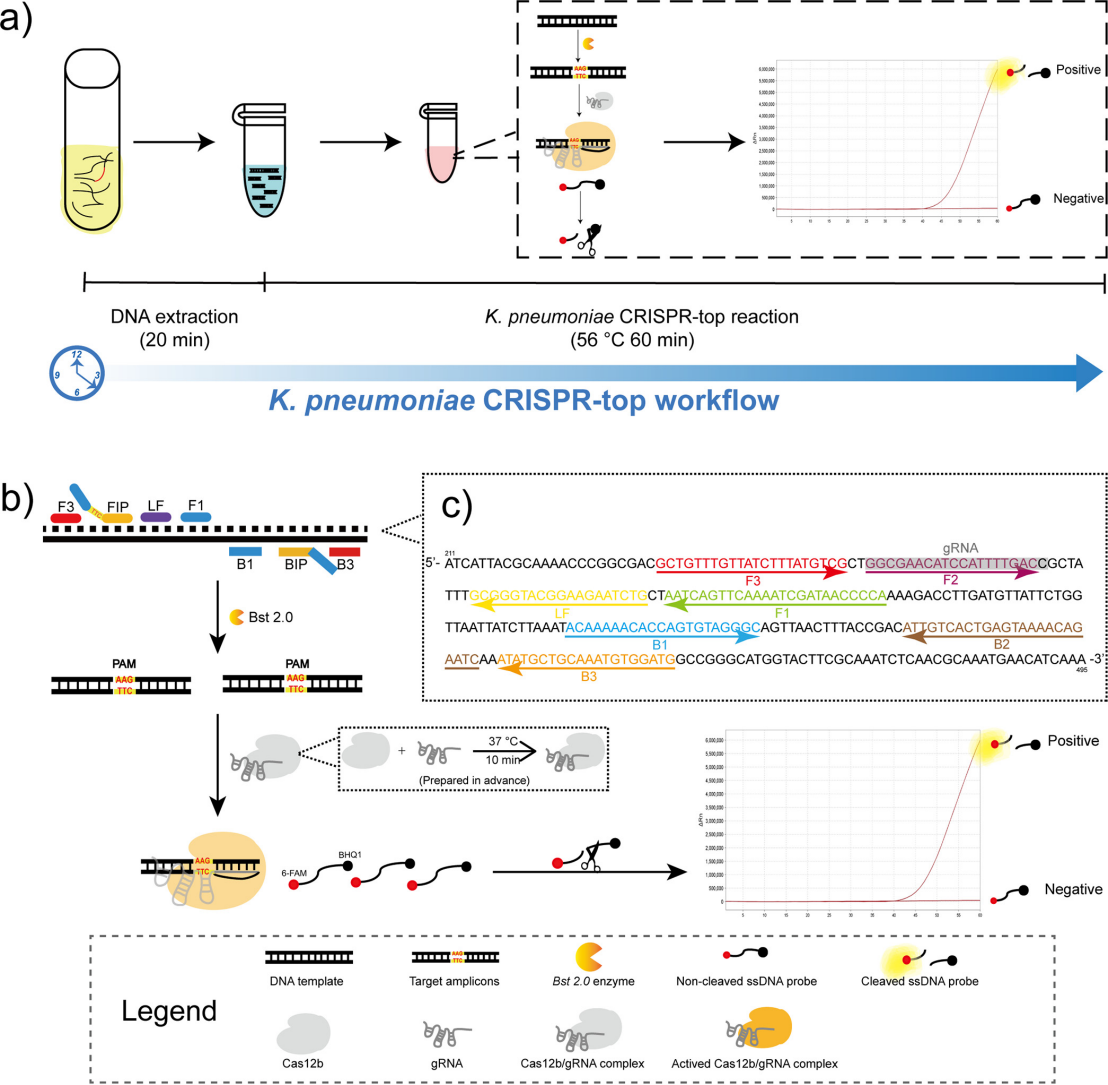
The principle of the K. pneumoniae CRISPR-top assay. (a) Schematic illustration of the K. pneumoniae CRISPR-top workflow. The sample-to-answer time of the K. pneumoniae CRISPR-top assay is less than 90 min. (b) The principle of the CRISPR-top reaction. (c) The primer set (Kp1) and gRNA design regions. A part of the *rcsA* sequence (211–495 bp) was shown. Right-pointing arrows and left-pointing arrows indicate sense and complementary strands, respectively.

In this report, we employed the CRISPR-top detection platform to establish a simple, rapid, and accurate method for K. pneumoniae detection and named it as K. pneumoniae CRISPR-top assay (Fig. 1). The capsular polysaccharide synthesis regulating gene *rcsA* was selected as the target sequence because of its specificity and conservation (15). The K. pneumoniae CRISPR-top assay was optimized after development and was verified using pure cultures. The limit of detection (LOD) of the present method was determined. Moreover, we compared the K. pneumoniae CRISPR-top assay with LAMP detection in terms of clinical diagnostic using clinical specimens to further clarify the advantages of the K. pneumoniae CRISPR-top assay.

## RESULTS

### Primer set selection and confirmation.

To screen out the most efficient primer set, LAMP amplification was performed using the three primers sets. All the three sets were tested under same reaction conditions. A real-time turbidimeter was employed to monitor the amplification products. K. pneumoniae ATCC 700603 genomic DNA (10 ng and 10 pg) was used as the positive control template, and deionized water (DW) was used as the negative control. Based on the results of LAMP tests (Fig. S1), set Kp1 was selected to establish the K. pneumoniae CRISPR-top assay because of the fastest amplification.

### Optimal reaction conditions of the K. pneumoniae CRISPR-top assay.

**(i) Optimal primer premixture volume.** Serial volumes of LAMP primer premixture (0.3–0.8 μL with 0.1 μL intervals) were used to determine the optimal primer concentration of the K. pneumoniae CRISPR-top assay. K. pneumoniae ATCC 700603 served as the positive template, and DW was used as the negative control. Finally, the reaction mixture with 0.4 μL LAMP primer premixture (work concentration 0.53 μM FIP and BIP, 0.27 μM LF, 0.13 μM F3 and B3) showed optimum detection results (Fig. S3).

**(ii) Optimal ssDNA probe concentration.** Different concentrations of ssDNA fluorescence probe (100 μM, 0.3 μL, 0.5 μL, 0.8 μL, 1 μL, and 1.5 μL) were examined to determine the optimal ssDNA probe concentration of the K. pneumoniae CRISPR-top assay. The positive reactions and the negative control were prepared as mentioned above. Finally, the reaction mixture with 0.5 μL ssDNA fluorescence probe (work concentration 2 μM) showed optimum detection results (Fig. S4).

**FIG 2:**
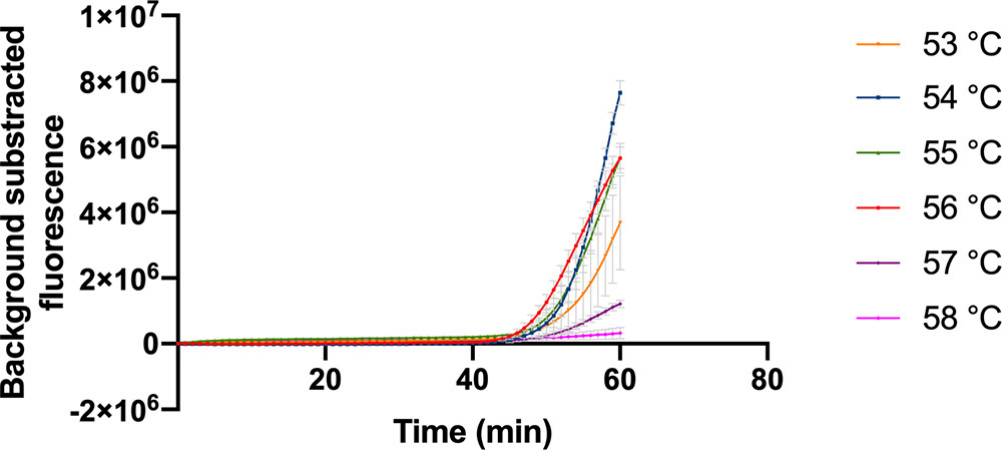
Optimal reaction temperature of the K. pneumoniae CRISPR-top assay. The reactions were performed under temperatures ranging from 53°C to 58°C with 1°C intervals. Error bars represented mean ± s.e.m. (*n* = 3 technical replicates). s.e.m., standard error of mean.

**(iii) Optimal reaction temperature**. The standard K. pneumoniae CRISPR-top reaction mixtures reacted under temperatures ranging from 53°C to 58°C with 1°C increments. As shown in Fig. 2, the most suitable reaction temperature was 56°C, because of the fastest reaction time and the relative high fluorescence value.

### Specificity of the K. pneumoniae CRISPR-top assay.

**FIG 3:**
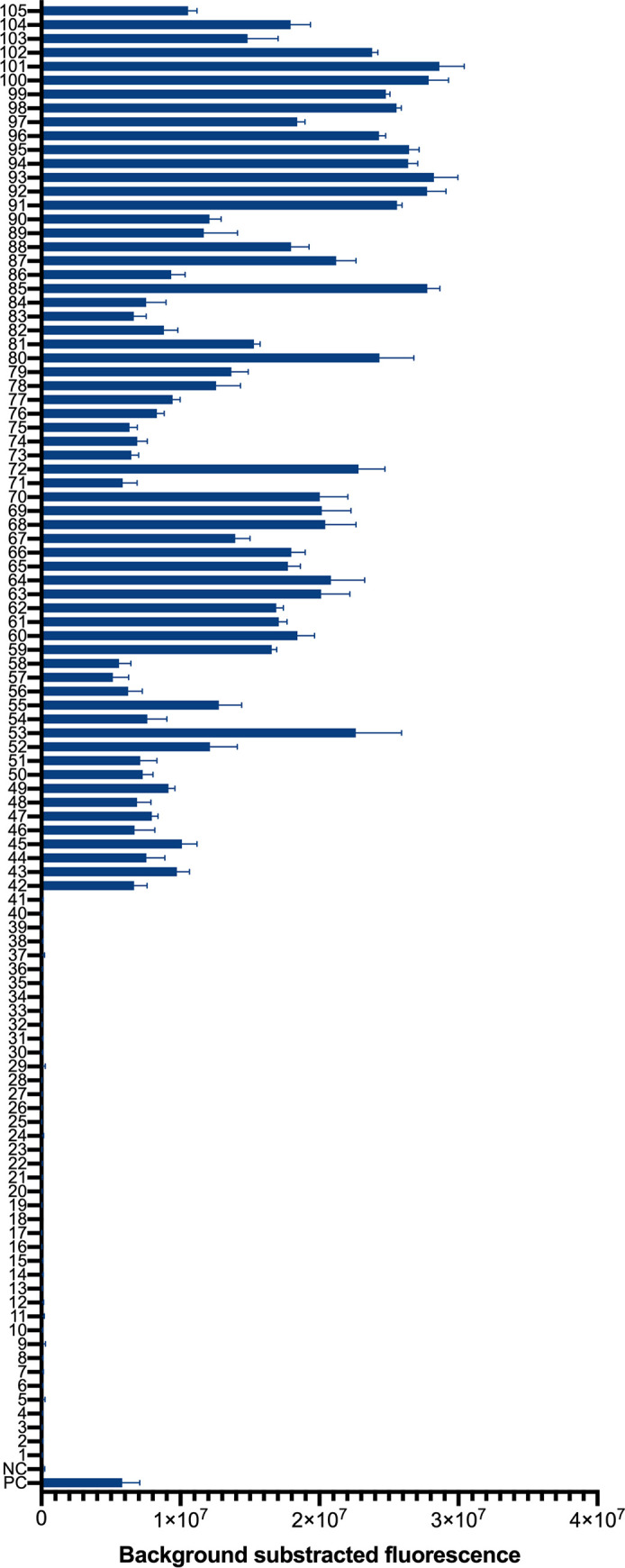
Specificity of the K. pneumoniae CRISPR-top assay. PC, positive control (K. pneumoniae ATCC 700603); NC, negative control (DW). 1–3, Klebsiella oxytoca; 4, Klebsiella aerogenes; 5–7, Staphylococcus aureus; 8, Staphylococcus haemolyticus; 9, Staphylococcus succinus; 10, Staphylococcus epidermidis; 11, Acinetobacter baumannii; 12, Acinetobacter *pizzeria*; 13, Acinetobacter junii; 14, Pseudomonas aeruginosa; 15, Haemophilus influenzae; 16, Moraxella catarrhalis; 17, Escherichia coli; 18, Mycobacterium tuberculosis; 19, Streptococcus pneumoniae; 20, Streptococcus oralis; 21, Streptococcus salivarius; 22, Streptococcus agalactiae; 23, Streptococcus pyogenes; 24, Streptococcus mitis; 25, Streptococcus suis; 26, Stenotrophomonas maltophilia; 27, Nocardia farcinica; 28, Nocardia cyriacigeorgica; 29, *Rhodococcus* sp.; 30, *Streptomyces* sp.; 31, Corynebacterium striatum; 32, Corynebacterium simulans; 33, Corynebacterium propinquum; 34, Corynebacterium aurimucosum; 35, Clostridium difficile; 36, Enterococcus faecalis; 37, Aeromonas caviae; 38, *Elizabethkingia anopheles*; 39, Ralstonia mannitolilytica; 40, *Rothia kristinae*; 41, Serratia marcescens; 42–105, K. pneumoniae clinical isolates (*n* = 3 technical replicates; bars represent mean ± s.e.m.).

K. pneumoniae ATCC 700603 and DW were used as the positive control and the negative control, respectively. In total, 105 strains, including 64 K. pneumoniae clinical isolates and 41 non- K. pneumoniae strains (Table S2) were tested by the K. pneumoniae CRISPR-top assay. As shown in Fig. 3, all the K. pneumoniae isolates tested showed positive and the non-K. pneumoniae strains and the negative control showed negative.

### LOD of the K. pneumoniae CRISPR-top assay.

**FIG 4:**
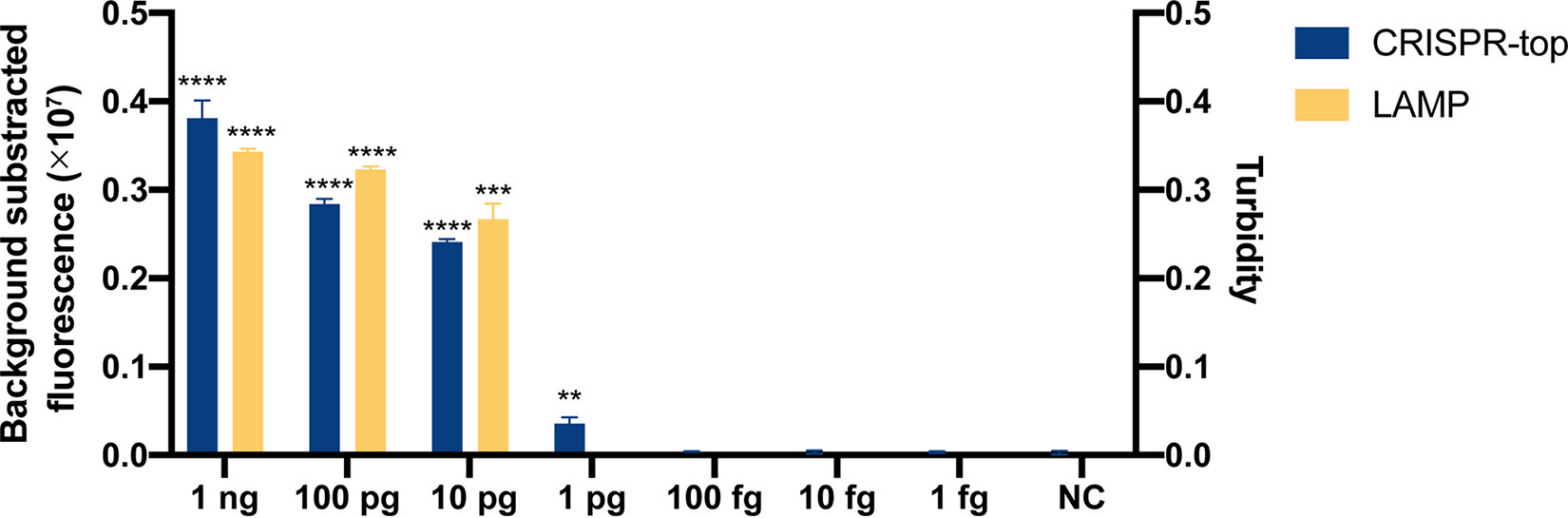
The limit of detections (LODs) of the K. pneumoniae CRISPR-top assay and the K. pneumoniae LAMP detection (*n* = 3 technical replicates, two-tailed Student's *t* test; bars represent mean ± s.e.m.). NC, negative control; **, *P* < 0.01; ***, *P* < 0.001; ****, *P* < 0.0001.

To determine the LOD of the K. pneumoniae CRISPR-top assay, genomic DNA was extracted from K. pneumoniae ATCC 700603 and diluted ranging from 1 ng/μL to 1 fg/μL at 10-fold intervals. As shown in Fig. 4, the LOD of the K. pneumoniae CRISPR-top assay was 1 pg pure genomic DNA (equivalent to 160 K. pneumoniae cells and 1.6 × 10^5^ CFU/mL for samples) per reaction, and the LOD of the LAMP detection was 10 pg pure genomic DNA, which was 10-fold higher than the K. pneumoniae CRISPR-top assay.

### Diagnostic performance of the K. pneumoniae CRISPR-top assay.

**FIG 5:**
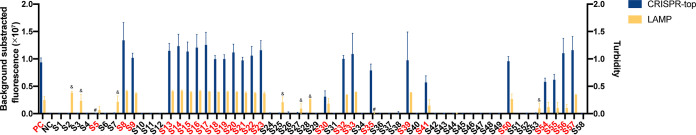
Clinical sample validation of the K. pneumoniae CRISPR-top assay and the K. pneumoniae LAMP detection. Culture was used as the gold standard and the numbers of the positive samples were in red (*n* = 3 technical replicates; bars represent mean ± s.e.m.). PC, positive control (K. pneumoniae ATCC 700603); NC, negative control (DW); &, false-positive results; #, false-negative results.

Of 58 clinical sputum specimens, K. pneumoniae strains were successfully isolated from 25 sputum samples and no other pathogen was identified in these samples. To take the result of culture as the gold standard, the sensitivities of the K. pneumoniae CRISPR-top assay and the LAMP detection were 96% (24/25) and 95.8% (23/24), respectively; and the specificities were 100% (33/33) and 79.4% (27/34), respectively. The positive predictive values of the K. pneumoniae CRISPR-top assay and the LAMP detection were 100% (24/24) and 76.7% (23/30), respectively; and the negative predictive values were 97.1% (33/34) and 96.4% (27/28), respectively. More details of detection using clinical samples are shown in Fig. 5.

## DISCUSSION

Klebsiella pneumoniae is a significant threat to global health, and the existing methods of K. pneumoniae detection are not suitable for rapid diagnosis. CRISPR-based diagnostics were one of seven technologies to watch in 2022 by Nature journal (24) and offered highly specific and ultra-sensitive tests. Compared to culture-based identification methods (e.g., biochemical identification and MALDI-TOF MS), CRISPR-based diagnostics are more timesaving. Compared to PCR-based tests, CRISPR-based diagnostics are able to perform isothermally with reduced dependency on specialized instruments. Compared to isothermal amplification (e.g., loop-mediated isothermal amplification, recombinase polymerase amplification), CRISPR-based diagnostics eliminate false positive results caused by primer cross-reaction, and show higher specificity. In this study, we employed a one-step, one-pot CRISPR-based nucleic acid detection platform (CRISPR-top) to develop the K. pneumoniae CRISPR-top assay for simple, rapid and accurate K. pneumoniae detection.

As expected, the K. pneumoniae CRISPR-top assay was extremely specific for K. pneumoniae detection and achieved 100% inclusivity and exclusivity applied to various pathogens (Table S2 and Fig. 3). In terms of clinical diagnosis, the K. pneumoniae CRISPR-top assay showed higher specificity and sensitivity compared with LAMP detection (Fig. 5), and no false-positive result was observed by our assay. The further increased reliability and precision in CRISPR-based detection could be owed to the target-dependent gRNA. Therefore, our data demonstrated the K. pneumoniae CRISPR-top assay can identify K. pneumoniae accurately.

Another advantage of the K. pneumoniae CRISPR-top assay is that it is simple and rapid. It only requires one fluid-handling step and is performed at a constant temperature (56°C) for 60 min. Without redundant liquid handling steps and manual operations, the process of detection is simplified, and the risk of cross-contamination is decreased. For results readout, a real-time fluorescence reader can be used, and visual detection can also be achieved using lateral flow biosensors and many small and portable blue light instruments (e.g., G500312, Blue Light Gel Imager, Sangon, China). Thus, an isothermal hot block or a temperature-controlled water bath and a portable blue light device or CRISPR test strips are enough for the K. pneumoniae CRISPR-top reaction and results readout. In addition, the K. pneumoniae CRISPR-top assay is low cost. A $500 USD portable heater supports 96 CRISPR-top reactions, and each reaction approximately costs $3.50 USD. A blue light device is also not expensive, and the device can be reused. Therefore, the K. pneumoniae CRISPR-top assay is appropriate for rapid detection of K. pneumoniae, especially for on-site testing or in resource-limited settings.

A limitation of the present method is that the LOD is relatively high (1 pg genomic DNA, equivalent to 160 K. pneumoniae cells and 1.6 × 10^5^ CFU/mL for samples), which may cause false negative results in infection with low dose. The relatively high LOD may be caused by relatively low amplification efficiency of the LAMP primer set in this study, which is inferred based on the results that the LOD of the K. pneumoniae CRISPR-top assay was 10-fold lower than the LAMP detection using the same primer set (Fig. 4). Moreover, the feasibility of this testing using bacterial suspension or samples without nucleic acid extraction was not tested in this study. Thus, a DNA extraction step is recommended for the K. pneumoniae CRISPR-top assay.

In conclusion, we established the K. pneumoniae CRISPR-top assay, which was a one-step and one-pot CRISPR-based method to detect K. pneumoniae simply, rapidly, and accurately at a constant temperature. The CRISPR-top assay showed higher specificity and sensitivity than the LAMP assay, and thus it can be a high-efficiency alternative tool for rapid and accurate diagnosis of K. pneumoniae infection, especially in resource-limited settings.

## MATERIALS AND METHODS

### Reagents and instruments.

Wizard Genomic DNA Purification kits were purchased and used to extract DNA (A1125; Promega, USA). DNA Isothermal Amplification kits (HT0600; HuiDeXin, China) and AapCas12b nuclease (HT100008; HuiDeXin, China) were purchased and used for LAMP and CRISPR-top assay. A real-time turbidimeter (Loopamp LA-320c; Eiken, Japan) was used to monitor the LAMP products. A real-time fluorescence qPCR instrument (QuantStudio 6 Flex; Applied Biosystems, USA) was used as the fluorescence reader. MALDI-TOF MS (microflex LRF; Bruker, Germany) was used to identify the isolated strains.

### Bacterial isolates, identification, and preparation of DNA templates.

K. pneumoniae reference strain ATCC 700603 was chosen to establish the K. pneumoniae CRISPR-top assay. Sixty-four K. pneumoniae clinical isolates isolated from different patients’ specimens and 41 non-K. pneumoniae strains purchased from international strain preservation center (standard strains) or collected from two hospitals in China (clinical isolates) were used to test the specificity of the K. pneumoniae CRISPR-top assay (Table S2). All the isolates mentioned above were stored in 20% (wt/vol) glycerol broth at −70°C before DNA extraction. Genomic DNA of isolates were extracted using Wizard Genomic DNA Purification kits according to the technical manual. The concentration of DNA of the isolates were greater than 10 ng/μL determined by a Nanodrop LITE spectrophotometer (Thermo Fisher, China). In the LOD examination, the genomic DNA of K. pneumoniae reference strain ATCC 700603 was serially diluted from 1 ng/μL to 1 fg/μL (10-fold intervals) after being quantified by the Nanodrop LITE instrument. All the clinical isolated K. pneumoniae were handled in a biosafety level 2 (BSL-2) laboratory.

### Primer and gRNA design.

Based on the sequence of *rcsA* (GenBank Gene ID: 7946097), three primer sets (Kp1–Kp3) were designed using PrimerExplorer V5 (http://primerexplorer.jp/lampv5e/index.html) and a PAM sequence (TTC) of AapCas12b was inserted at the linker region (between F1c and F2) of each forward inner primer (FIP) according to the strategy of the CRISPR-top platform (Table 1 and Table S1) (23). Oligo Analyzer version 3.1 (https://eu.idtdna.com/calc/analyzer) was used to check the secondary structure of primers. The guide RNA (gRNA) and the single strand DNA (ssDNA) fluorescence probe (6-FAM/BHQ1 labeled) were designed based on the principle of the CRISPR-top platform (23). The details on primers and gRNA design are shown in Table 1, Table S1, Fig. 1, and Fig. S2. The LAMP primers and the ssDNA fluorescence probe were synthesized and purified at HPLC purification grade by Sangon Biotech. Co., Ltd. (Shanghai, China). The gRNA was synthesized and purified at HPLC purification grade by GenScript Biotech. Co., Ltd. (Nanjing, China).

**TABLE 1:** Primers, gRNA, and probe used to establish the K. pneumoniae CRISPR-top assay

Primer	Type	Sequence (5′–3′)^*a*^	Length^*b*^
Kp1-F3	Forward outer primer	GCTGTTTGTTATCTTTATGTCG	22 nt
Kp1-B3	Backward outer primer	CATCCACATTTGCAGCATAT	20 nt
Kp1-FIP	Forward inner primer	TGGGGTTATCGATTTTGAACTGATT-**TTC**-GGCGAACATCCATTTTGAC	47 nt
Kp1-BIP	Backward inner primer	ACAAAAACACCAGTGTAGGGC-GATTCTGTTTTACTCAGTGACAAT	45 nt
Kp1-LF	Loop forward primer	CAGATTCTTCCGTACCCGC	19 nt
gRNA	Guide RNA	GUCUAGAGGACAGAAUUUUUCAACGGGUGUGCCAAUGGCCACUUUCCAGGUGGCAAAGCCCGUUGAGCUUCUCAAAUCUGAGAAGUGGCACGGCGAACAUCCAUUUUGACC	111 nt
Probe	SsDNA fluorescence probe	FAM-TTATTATTAT-BHQ1	10 nt

a The inserted PAM site (TTC) is in bold.

*^b^*nt, nucleotide.

### Standard K. pneumoniae CRISPR-top assay.

According to the protocol of CRISPR-top, the reaction was performed in a final volume of 25 μL (23). Each reaction contained 12.5 μL 2× isothermal reaction buffer, 1 μL *Bst* 2.0 DNA polymerase (8U), 0.4 μL LAMP primer premixture, 3.5 μL AapCas12b-gRNA complex, 0.5 μL ssDNA fluorescence probe (100 μM), 1 μL DNA template, and DNase/RNase-free deionized water (DW) up to 25 μL. The DNA template was replaced by DW in the negative control reaction. The LAMP primers were diluted to 100 μM and premixed in advance. The LAMP primer premixture contained 40 μL each of FIP and BIP, 20 μL each of LF and/or LB, and 10 μL each of F3 and B3. The AapCas12b-gRNA complex mixture was prepared using 15 pmol AapCas12b and 2 μL gRNA (10 μM) in Cas12b buffer and was incubated at 37°C for 10 min. The complex was used immediately after preparation. The CRISPR-top reaction was monitored by the fluorescence reader at a constant temperature for 1 h.

### LAMP detection.

A 25 μL LAMP reaction volume contained 12.5 μL 2× isothermal reaction buffer, 1 μL *Bst* 2.0 DNA polymerase (8U), 0.4 μL LAMP primer premixture, 1 μL DNA template, and DNase/RNase-free deionized water (DW) up to 25 μL. The LAMP reaction was conducted at 56°C for 1 h using a real-time turbidimeter containing a thermo block (Loopamp LA-320c; Eiken, Japan), and the turbidity of the reaction mixture was captured by the turbidimeter in real time.

### Specificity and LOD of the K. pneumoniae CRISPR-top assay.

To examine the specificity of the K. pneumoniae CRISPR-top assay, DNA templates extracted from 64 K. pneumoniae clinical isolates and 41 non- K. pneumoniae strains (Table S2) were tested. K. pneumoniae ATCC 700603 served as the positive control and DW as the negative control. To determine the LOD of the K. pneumoniae CRISPR-top assay, 10-fold serial dilutions of K. pneumoniae ATCC 700603 genomic DNA (1 ng/μL to 1 fg/μL) were prepared and 1 μL aliquots of DNA templates were used in each reaction. A negative control (DW) was tested in parallel. The LOD of LAMP detection was determined using the same DNA templates with CRISPR-top. The experiments were repeated three times.

### Application of the K. pneumoniae CRISPR-top assay to clinical samples.

A total of 58 clinical sputum samples from patients with respiratory symptoms were collected by two hospitals in China to validate the K. pneumoniae CRISPR-top assay. One milliliter sputum samples were digested by 4% sodium hydroxide solution followed by DNA extraction using DNA purification kits. The K. pneumoniae CRISPR-top assay was compared with the LAMP detection using the same DNA templates from clinical samples. The experiments were repeated three times. The results of culture were used as the gold standard to evaluate the K. pneumoniae CRISPR-top assay and the LAMP detection in clinical diagnostic. The sputum samples were inoculated on Columbia blood agar plates, chocolate agar plates and eosin-methylene blue agar plates and incubated at 35°C for 24 to 72 h. Then, the isolated colonies were identified by MALDI-TOF MS.

### Ethics statement.

This study was approved by the Research Ethics Committee of National Institute for Communicable Disease Control and Prevention, Chinese Center for Disease Control and Prevention (no. ICDC-2019015). All experiments were performed according to relevant regulations.

## References

[1] Choby JE, Howard-Anderson J, Weiss DS. 2020. Hypervirulent Klebsiella pneumoniae - clinical and molecular perspectives. J Intern Med 287:283–300. doi:10.1111/joim.13007. 31677303 PMC7057273

[2] Pitout JD, Nordmann P, Poirel L. 2015. Carbapenemase-producing Klebsiella pneumoniae, a key pathogen set for global nosocomial dominance. Antimicrob Agents Chemother 59:5873–5884. doi:10.1128/AAC.01019-15. 26169401 PMC4576115

[3] Li Z-J, Zhang H-Y, Ren L-L, Lu Q-B, Ren X, Zhang C-H, Wang Y-F, Lin S-H, Zhang X-A, Li J, Zhao S-W, Yi Z-G, Chen X, Yang Z-S, Meng L, Wang X-H, Liu Y-L, Wang X, Cui A-L, Lai S-J, Jiang T, Yuan Y, Shi L-S, Liu M-Y, Zhu Y-L, Zhang A-R, Zhang Z-J, Yang Y, Ward MP, Feng L-Z, Jing H-Q, Huang L-Y, Xu W-B, Chen Y, Wu J-G, Yuan Z-H, Li M-F, Wang Y, Wang L-P, Fang L-Q, Liu W, Hay SI, Gao GF, Yang W-Z, Yang W-Z, Gao GF, Li Z-J, Wang L-P, Ren X, Wang Y-F, The Chinese Centers for Disease Control and Prevention (CDC) Etiology of Respiratory Infection Surveillance Study Team, et al. 2021. Etiological and epidemiological features of acute respiratory infections in China. Nat Commun 12:5026. doi:10.1038/s41467-021-25120-6. 34408158 PMC8373954

[4] Navon-Venezia S, Kondratyeva K, Carattoli A. 2017. Klebsiella pneumoniae: a major worldwide source and shuttle for antibiotic resistance. FEMS Microbiol Rev 41:252–275. doi:10.1093/femsre/fux013. 28521338

[5] Marr CM, Russo TA. 2019. Hypervirulent Klebsiella pneumoniae: a new public health threat. Expert Rev Anti Infect Ther 17:71–73. doi:10.1080/14787210.2019.1555470. 30501374 PMC6349525

[6] Moradigaravand D, Martin V, Peacock SJ, Parkhill J. 2017. Evolution and epidemiology of multidrug-resistant Klebsiella pneumoniae in the United Kingdom and Ireland. mBio 8. doi:10.1128/mBio.01976-16. PMC5358916 28223459

[7] Abdeta A, Bitew A, Fentaw S, Tsige E, Assefa D, Lejisa T, Kefyalew Y, Tigabu E, Evans M. 2021. Phenotypic characterization of carbapenem non-susceptible gram-negative bacilli isolated from clinical specimens. PLoS One 16:e0256556. doi:10.1371/journal.pone.0256556. 34855767 PMC8638961

[8] Keshta AS, Elamin N, Hasan MR, Pérez-López A, Roscoe D, Tang P, Suleiman M. 2021. Evaluation of rapid immunochromatographic tests for the direct detection of extended spectrum beta-lactamases and carbapenemases in Enterobacterales isolated from positive blood cultures. Microbiol Spectr 9:e0078521. doi:10.1128/Spectrum.00785-21. 34878297 PMC8653814

[9] Mari-Almirall M, et al. 2021. Clonal spread and intra- and inter-species plasmid dissemination associated with Klebsiella pneumoniae carbapenemase-producing Enterobacterales during a hospital outbreak in Barcelona, Spain. Front Microbiol 12:781127. doi:10.3389/fmicb.2021.781127. 34867923 PMC8637019

[10] Huang Y, Li J, Wang Q, Tang K, Li C. 2022. Rapid detection of KPC-producing Klebsiella pneumoniae in China based on MALDI-TOF MS. J Microbiol Methods 192:106385. doi:10.1016/j.mimet.2021.106385. 34843862

[11] Liu Y, Liu C, Zheng W, Zhang X, Yu J, Gao Q, Hou Y, Huang X. 2008. PCR detection of Klebsiella pneumoniae in infant formula based on 16S-23S internal transcribed spacer. Int J Food Microbiol 125:230–235. doi:10.1016/j.ijfoodmicro.2008.03.005. 18579248

[12] Barbier E, Rodrigues C, Depret G, Passet V, Gal L, Piveteau P, Brisse S. 2020. The ZKIR Assay, a real-time PCR method for the detection of Klebsiella pneumoniae and closely related species in environmental samples. Appl Environ Microbiol 86:e02711-19. doi:10.1128/AEM.02711-19. PMC7082575 32005732

[13] Si Y, Zhang T, Chen N, Cheng Y, Wang L, Yuan J, Li G, Zong M, Sui G, Fan L. 2021. A LAMP-based system for rapid detection of eight common pathogens causing lower respiratory tract infections. J Microbiol Methods 190:106339. doi:10.1016/j.mimet.2021.106339. 34592373

[14] Chen Y, et al. 2021. Rapid detection of Clostridium botulinum in food using loop-mediated isothermal amplification (LAMP). Int J Environ Res Public Health 18:4401. doi:10.3390/ijerph18094401. 33919101 PMC8122632

[15] Dong D, Liu W, Li H, Wang Y, Li X, Zou D, Yang Z, Huang S, Zhou D, Huang L, Yuan J. 2015. Survey and rapid detection of Klebsiella pneumoniae in clinical samples targeting the rcsA gene in Beijing, China. Front Microbiol 6:519. 26052327 10.3389/fmicb.2015.00519 PMC4440914

[16] Li S-Y, Cheng Q-X, Wang J-M, Li X-Y, Zhang Z-L, Gao S, Cao R-B, Zhao G-P, Wang J. 2018. CRISPR-Cas12a-assisted nucleic acid detection. Cell Discov 4:20. doi:10.1038/s41421-018-0028-z. 29707234 PMC5913299

[17] Chen JS, Ma E, Harrington LB, Da Costa M, Tian X, Palefsky JM, Doudna JA. 2018. CRISPR-Cas12a target binding unleashes indiscriminate single-stranded DNase activity. Science 360:436–439. doi:10.1126/science.aar6245. 29449511 PMC6628903

[18] Gootenberg JS, Abudayyeh OO, Lee JW, Essletzbichler P, Dy AJ, Joung J, Verdine V, Donghia N, Daringer NM, Freije CA, Myhrvold C, Bhattacharyya RP, Livny J, Regev A, Koonin EV, Hung DT, Sabeti PC, Collins JJ, Zhang F. 2017. Nucleic acid detection with CRISPR-Cas13a/C2c2. Science 356:438–442. doi:10.1126/science.aam9321. 28408723 PMC5526198

[19] Wu H, Chen Y, Yang Q, Peng C, Wang X, Zhang M, Qian S, Xu J, Wu J. 2021. A reversible valve-assisted chip coupling with integrated sample treatment and CRISPR/Cas12a for visual detection of Vibrio parahaemolyticus. Biosens Bioelectron 188:113352. doi:10.1016/j.bios.2021.113352. 34038837

[20] Chen Y, Mei Y, Zhao X, Jiang X. 2020. Reagents-loaded, automated assay that integrates recombinase-aided amplification and Cas12a nucleic acid detection for a point-of-care test. Anal Chem 92:14846–14852. doi:10.1021/acs.analchem.0c03883. 33064442

[21] Wang B, Wang R, Wang D, Wu J, Li J, Wang J, Liu H, Wang Y. 2019. Cas12aVDet: a CRISPR/Cas12a-based platform for rapid and visual nucleic acid detection. Anal Chem 91:12156–12161. doi:10.1021/acs.analchem.9b01526. 31460749

[22] Wang R, Qian C, Pang Y, Li M, Yang Y, Ma H, Zhao M, Qian F, Yu H, Liu Z, Ni T, Zheng Y, Wang Y. 2021. opvCRISPR: one-pot visual RT-LAMP-CRISPR platform for SARS-cov-2 detection. Biosens Bioelectron 172:112766. doi:10.1016/j.bios.2020.112766. 33126177 PMC7586109

[23] Li S, Huang J, Ren L, Jiang W, Wang M, Zhuang L, Zheng Q, Yang R, Zeng Y, Luu LDW, Wang Y, Tai J. 2021. A one-step, one-pot CRISPR nucleic acid detection platform (CRISPR-top): application for the diagnosis of COVID-19. Talanta 233:122591. doi:10.1016/j.talanta.2021.122591. 34215080 PMC8197615

[24] Eisenstein M. 2022. Seven technologies to watch in 2022. Nature 601:658–661. doi:10.1038/d41586-022-00163-x. 35079149

