# Supplementary material for: One-Pot Isothermal LAMP-CRISPR-Based Assay for Klebsiella pneumoniae Detection
Source: Microbiol Spectr. 2022 Jul 20;10(4):e01545-22. doi: 10.1128/spectrum.01545-22 (PMC9430409; doi:10.1128/spectrum.01545-22)
Supplement: Supplemental file 1 — Supplemental material. Download spectrum.01545-22-s0001.pdf, PDF file, 0.7 MB [file spectrum.01545-22-s0001.pdf]

Supplementary materials for one-pot, isothermal LAMP-CRISPR-based assay for *Klebsiella pneumoniae* detection

Table S1. Alternative LAMP primers designed in this study.

Primer	Type	Sequence (5'-3') [a]	Length [b]
Kp2-F3	Forward outer primer	CTATACCCGGTTGGGATTG	19 nt
Kp2-B3	Backward outer primer	AATGCCATTACTTTCATCATCA	22 nt
Kp2-FIP	Forward inner primer	AGCACTGTTGACCTCAACGA-**TTC**-ACGGGATATCTGACCAGTC	42 nt
Kp2-BIP	Backward inner primer	GCGGATCTGCAGAAACACTG-CACGAAACAGTCTTCATTCA	40 nt
Kp2-LF	Loop forward primer	TCCTGTTTTTTAATTCCCC	19 nt
Kp2-LB	Loop backward primer	CGTCGTGTTGCCCGGCGGT	19 nt
Kp3-F3	Forward outer primer	ATTGTCACTGAGTAAAACAGAA	22 nt
Kp3-B3	Backward outer primer	AGGTGATGTTTTCGGTCAG	19 nt
Kp3-FIP	Forward inner primer	GCTTTGATGTTCATTTGCGTTGAG-**TTC**-TGCTGCAAATGTGGATGG	45 nt
Kp3-BIP	Backward inner primer	AAGACGGTATCGTCGCATAAA-GAACGATATGATAAATCACCTGC	44 nt
Kp3-LF	Loop forward primer	TGCGAAGTACCATGCCCGG	19 nt

[a] The inserted PAM site (TTC) was in bold. [b] nt, nucleotide.

Table S2. Bacterial strains used in this study.

Bacteria	Strain type (Strain ID)	No. of strains
Klebsiella pneumoniae	Reference strain (ATCC 700603)	1
	Isolated strain	64
Klebsiella oxytoca	Isolated strain	3
Klebsiella aerogenes	Isolated strain	1
Staphylococcus aureus	Reference strain (ATCC 29213)	1
	Isolated strain	2
Staphylococcus haemolyticus	Isolated strain	1
Staphylococcus succinus	Isolated strain	1
Staphylococcus epidermidis	Isolated strain	1
Acinetobacter baumannii	Isolated strain	1
Acinetobacter pizzeri	Isolated strain	1
Acinetobacter junii	Isolated strain	1
Pseudomonas aeruginosa	Reference strain (ATCC 27853)	1
Haemophilus influenzae	Reference strain (ATCC 49247)	1
Moraxella catarrhalis	Isolated strain	1
Escherichia coli	Reference strain (ATCC 25922)	1
Mycobacterium tuberculosis	Reference strain (H37Rv)	1
Streptococcus pneumoniae	Reference strain (ATCC 49619)	1
Streptococcus oralis	Isolated strain	1
Streptococcus salivarius	Reference strain (K12)	1
Streptococcus agalactiae	Isolated strain	1
Streptococcus pyogenes	Reference strain (CGMC 48848)	1
Streptococcus mitis	Isolated strain	1
Streptococcus suis	Reference strain (GZ1)	1
Stenotrophomonas maltophilia	Isolated strain	1
Nocardia farcinica	Reference strain (IFM 10152)	1
Nocardia cyriacigeorgica	Reference strain (DSM 40350)	1
Rhodococcus sp.	Isolated strain	1
Streptomyces sp.	Isolated strain	1
Corynebacterium striatum	Reference strain (ATCC 43751)	1
Corynebacterium simulans	Isolated strain	1
Corynebacterium propinquum	Isolated strain	1
Corynebacterium aurimucosum	Isolated strain	1
Clostridium difficile	Reference strain (ATCC BAA-1803)	1
Enterococcus faecalis	Reference strain (ATCC 29212)	1
Aeromonas caviae	Isolated strain	1
Elizabethkingia anophelis	Isolated strain	1
Ralstonia mannitolilytica	Isolated strain	1
Rothia kristinae	Isolated strain	1
Serratia marcescens	Isolated strain	1

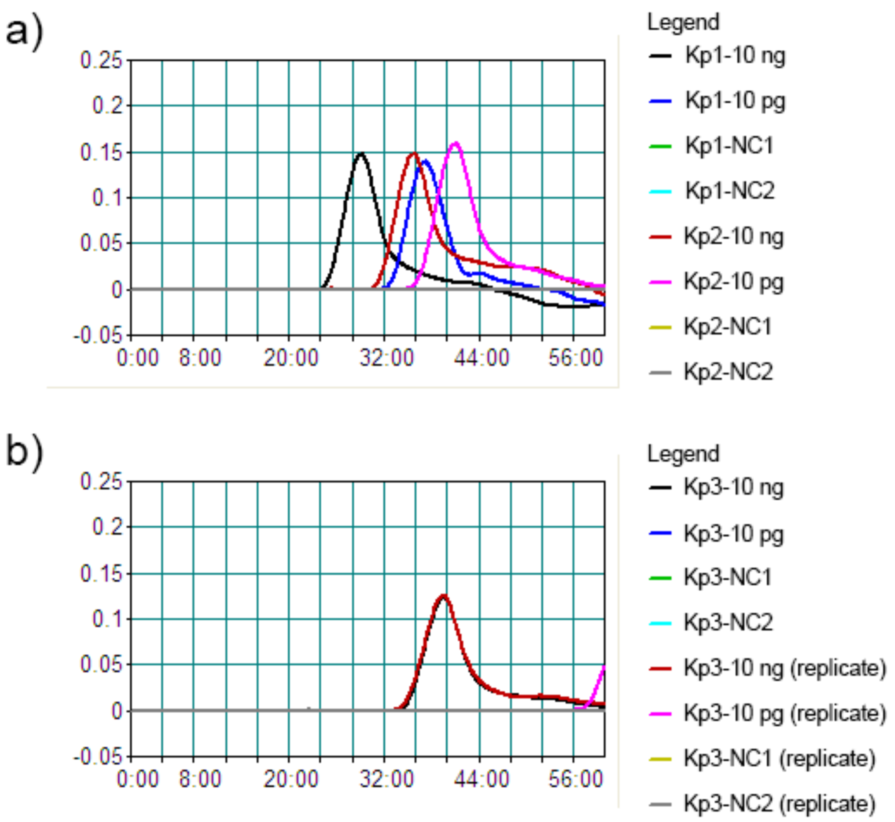

Figure S1. Primer sets screening and confirmation. LAMP detection was conducted at 59 °C for 1 h. **a)** Primer set Kp1 and Kp2. **b)** Primer set Kp3.

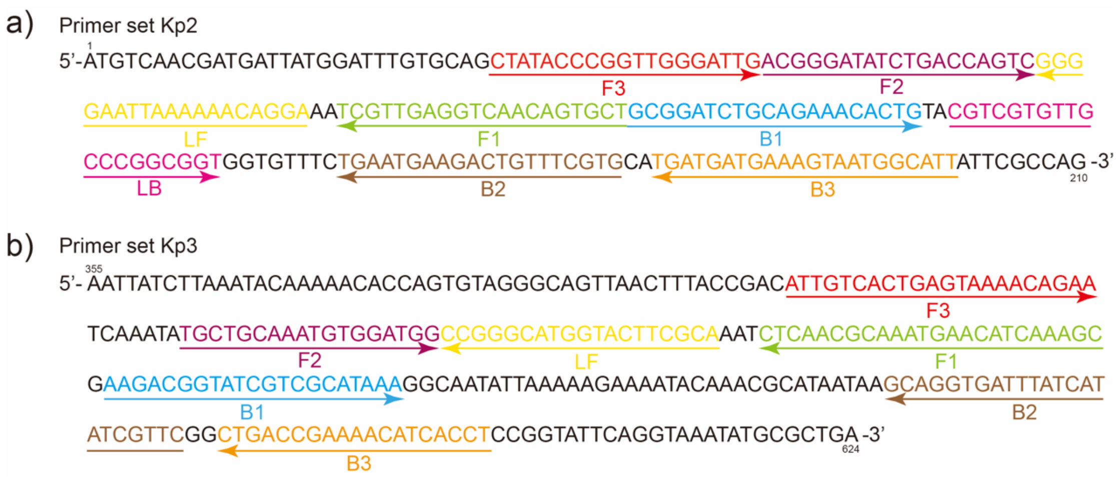

Figure S2. The primer set Kp2 and Kp3 design regions. A part of the *rcsA* sequence was shown. Right-pointing arrows and left-pointing arrows indicate sense and complementary strands respectively. **a)** Primer set Kp2. **b)** Primer set Kp3.

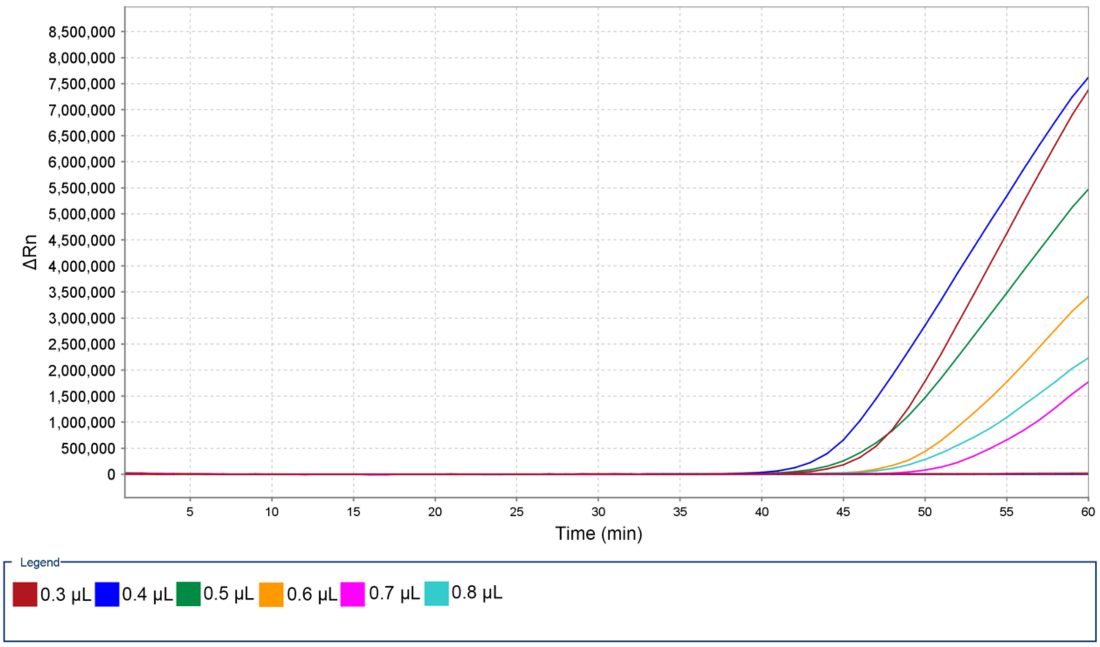

Figure S3. Optimal primer pre-mixture volume of the *K. pneumoniae* CRISPR-top assay. *K. pneumoniae* ATCC 700603 genomic DNA and DW were used as the templates of the positive control and the negative control respectively. PC, positive control; NC, negative control.

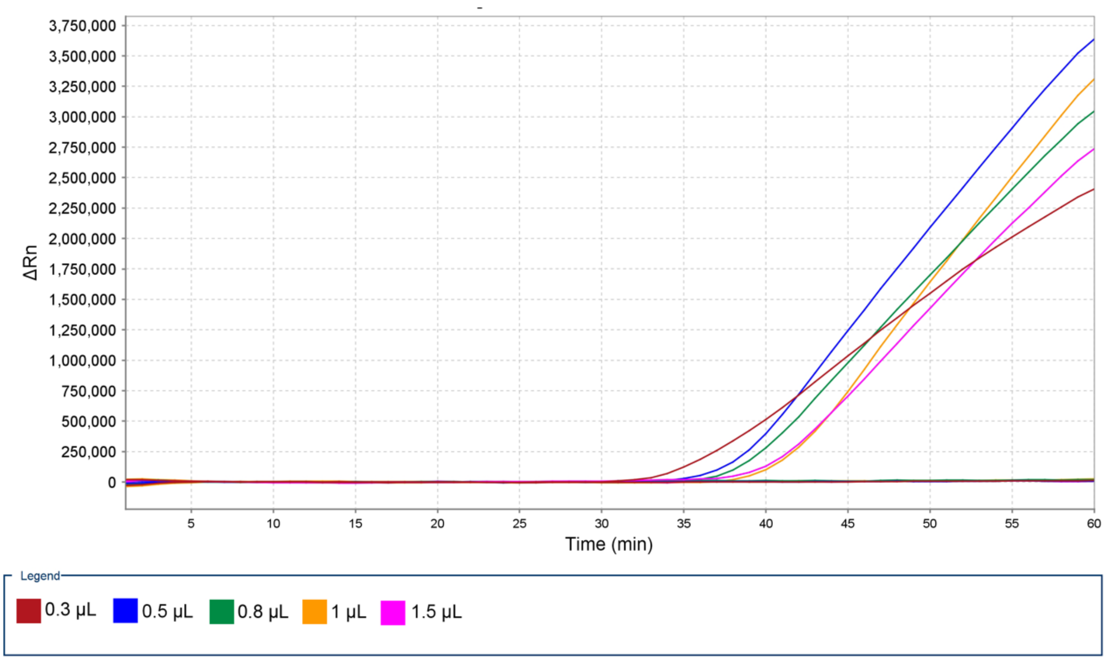

Figure S4. Optimal ssDNA probe concentration of the *K. pneumoniae* CRISPR-top assay. *K. pneumoniae* ATCC 700603 genomic DNA and DW were used as the templates of the positive control and the negative control respectively. PC, positive control; NC, negative control.
